# Supplementary material for: Host- plasmid network structure in wastewater is linked to antimicrobial resistance genes
Source: Nat Commun. 2024 Jan 16;15:555. doi: 10.1038/s41467-024-44827-w (PMC10791616; doi:10.1038/s41467-024-44827-w)
Supplement: Supplementary file 1 — Supplementary Information [file 41467_2024_44827_MOESM1_ESM.pdf]

Supplementary material

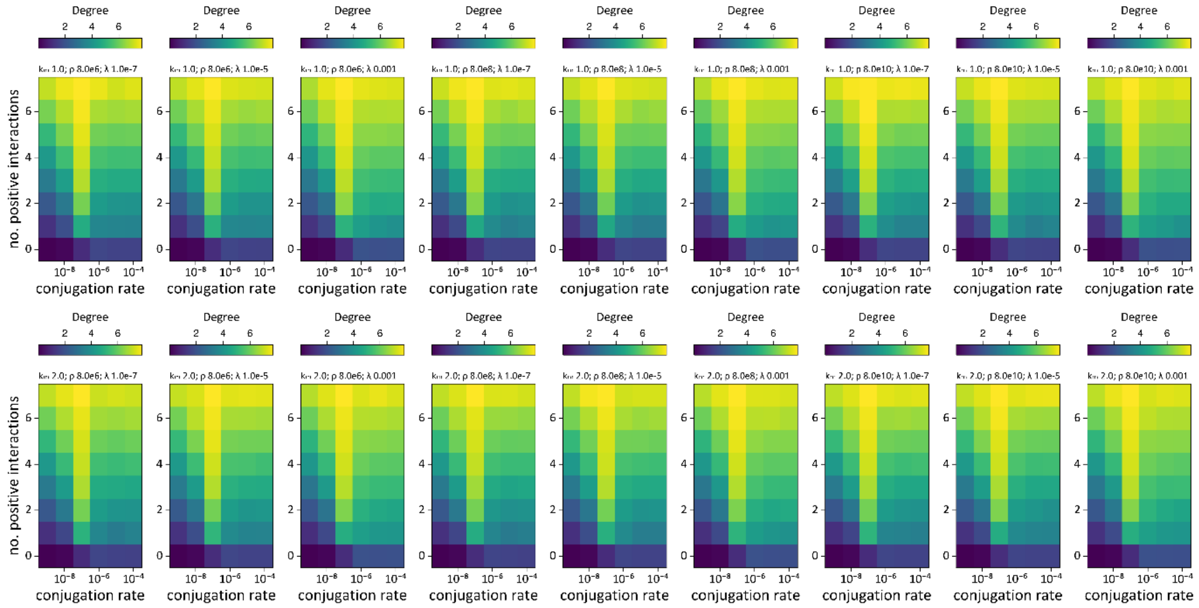

Figure S1. Additional plasmid population dynamics model results with a single beneficial plasmid. Mean plasmid degree in the network (number of hosts with at least 1% infection rate for a given plasmid) at equilibrium, for a range of conjugation rates. Results shown are for simulations with 8 hosts and 3 plasmids, where only a single plasmid is capable of positive interactions. Panels are labelled with parameter values: ρ is the mean of the Normal distribution from which all ρ^i values were drawn, Km and λ are the half-saturation constant and rate of segregational loss of plasmids that was shared between all strains.

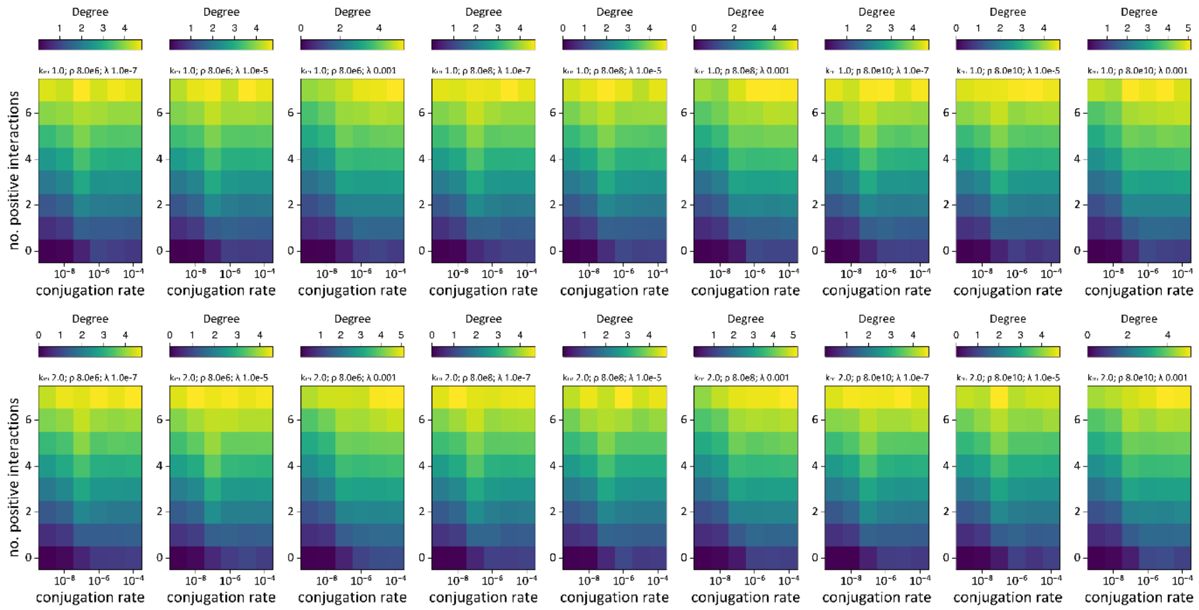

Figure S2. Additional plasmid population dynamics model results where all plasmids may be beneficial. Mean plasmid degree in the network (number of hosts with at least 1% infection rate for a given plasmid) at equilibrium, for a range of conjugation rates. Results shown are for simulations with 8 hosts and 3 plasmids, where all plasmids may or may not interact positively (increase host fitness) with a set of hosts. Panels are labelled with parameter values: ρ is the mean of the Normal distribution from which all ρ^i values were drawn, Km and λ are the half-saturation constant and rate of segregational loss of plasmids that was shared between all strains.

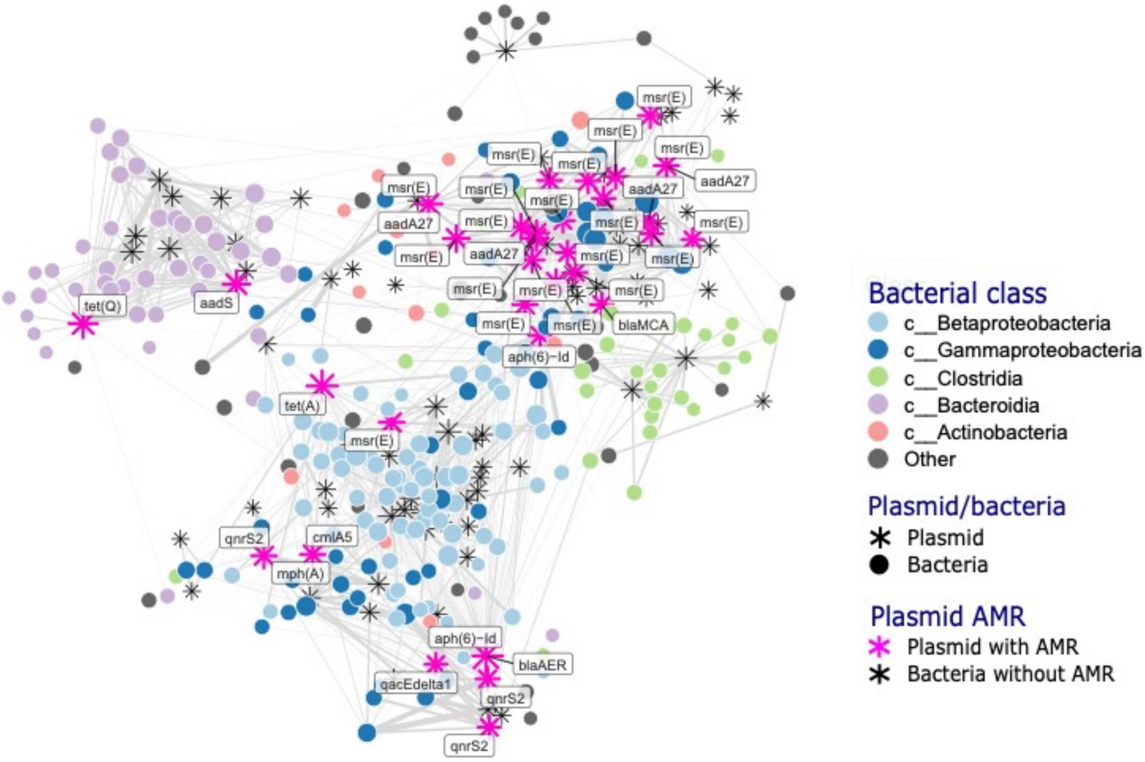

Figure S3. Full bacteria-plasmid network, including the names of AMR genes associated with each AMR plasmid. Data to replicate this figure is contained in the source data file.

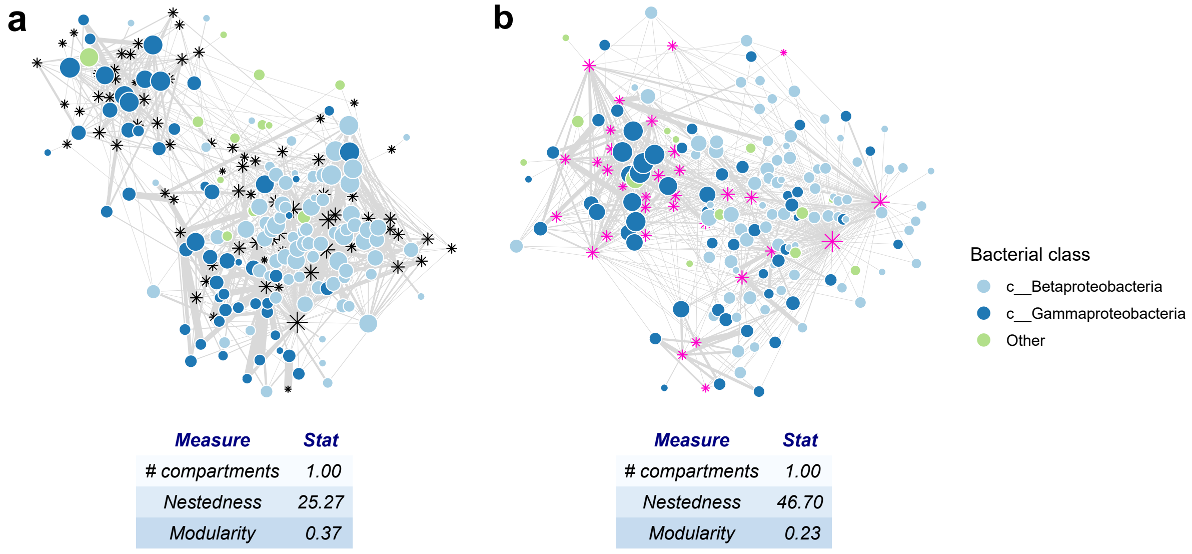

Figure S4. Proteobacteria sub-network. Sub-networks and network statistics when retaining only a) plasmids without AMR genes and b) plasmids with AMR genes. Stars represent plasmids and circles MAGs, with AMR plasmids highlighted in pink. Widths of the edges represent the strength of the Hi-V connection. Data to replicate this figure is contained in the source data file.

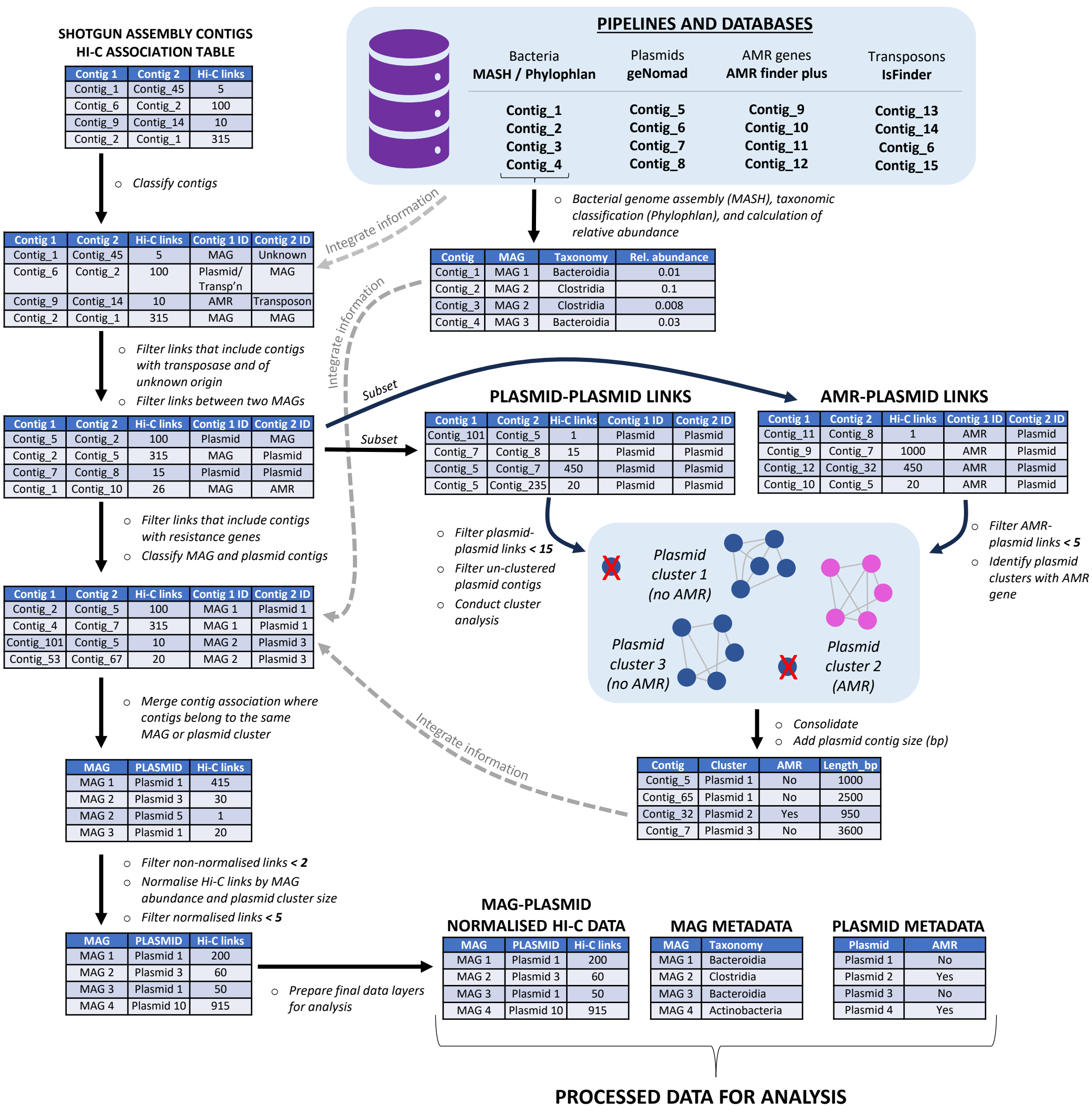

Figure S5. Flowchart visualising the Hi-C processing workflow prior to data analysis. Visual representation of the major steps performed for the processing of Hi-C data.

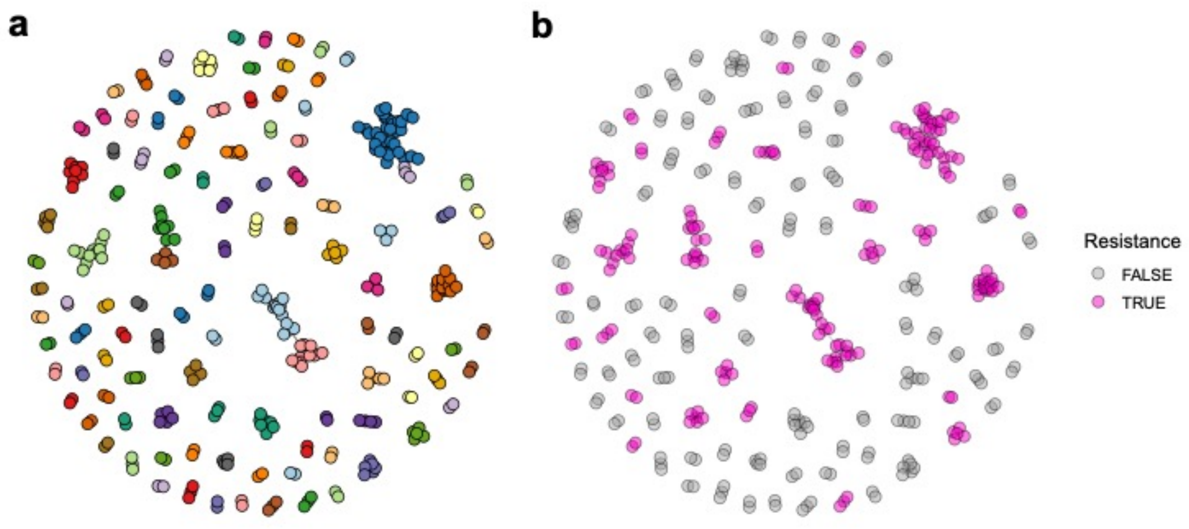

Figure S6. Putative plasmid clusters (379 contigs clustered through Hi-C linkage patterns into 109 putative plasmids) that were retained for analysis. Each node represents a contig identified as a plasmid fragment by geNomad. Nodes are coloured by a) its cluster membership based on the walktrap method, and b) whether it is associated with AMR (pink). Data to replicate this figure is contained in the source data file.
