# Supplementary material for: Host- plasmid network structure in wastewater is linked to antimicrobial resistance genes
Source: Nat Commun. 2024 Jan 16;15:555. doi: 10.1038/s41467-024-44827-w (PMC10791616; doi:10.1038/s41467-024-44827-w)
Supplement: Supplementary file 3 — Description of Additional Supplementary Files [file 41467_2024_44827_MOESM3_ESM.pdf]

Description of Additional Supplementary Files

Supplementary Data 1. Proximeta metadata for each MAG included in this study (cluster_id), including its relative abundance, completeness, marker gene overrepresentation, mash marker lineage, mash hashes, likely taxonomy based on MASH assignment (mash_reference), mash p-value, genome size and GC content (gc%). The MASH p-value is calculated using the MinHash technique. The p-value represents the probability of obtaining a Mash distance as extreme as the one observed, assuming the null hypothesis that the sequences being compared come from the same genome. Further the table contains the number of contigs, n50, mean contig length, kmer coherence and the taxonomy obtained by Phylophlan.
