# Supplementary material for: Host- plasmid network structure in wastewater is linked to antimicrobial resistance genes
Source: Nat Commun. 2024 Jan 16;15:555. doi: 10.1038/s41467-024-44827-w (PMC10791616; doi:10.1038/s41467-024-44827-w)
Supplement: Supplementary file 5 — Reporting Summary [file 41467_2024_44827_MOESM5_ESM.pdf]

nature research

Corresponding author(s): *Double-blind peer review submissions: write DBPR and your manuscript number here instead of author names.*

Last updated by author(s): *YYYY-MM-DD*

Reporting Summary

Nature Research wishes to improve the reproducibility of the work that we publish. This form provides structure for consistency and transparency in reporting. For further information on Nature Research policies, see our Editorial Policies and the Editorial Policy Checklist.

Statistics

For all statistical analyses, confirm that the following items are present in the figure legend, table legend, main text, or Methods section.

n/a	Confirmed	
☐	☒	The exact sample size (*n*) for each experimental group/condition, given as a discrete number and unit of measurement
☐	☒	A statement on whether measurements were taken from distinct samples or whether the same sample was measured repeatedly
☒	☐	The statistical test(s) used AND whether they are one- or two-sided<br>*Only common tests should be described solely by name; describe more complex techniques in the Methods section.*
☒	☐	A description of all covariates tested
☒	☐	A description of any assumptions or corrections, such as tests of normality and adjustment for multiple comparisons
☒	☐	A full description of the statistical parameters including central tendency (e.g. means) or other basic estimates (e.g. regression coefficient) AND variation (e.g. standard deviation) or associated estimates of uncertainty (e.g. confidence intervals)
☒	☐	For null hypothesis testing, the test statistic (e.g. *F*, *t*, *r*) with confidence intervals, effect sizes, degrees of freedom and *P* value noted<br>*Give P values as exact values whenever suitable.*
☒	☐	For Bayesian analysis, information on the choice of priors and Markov chain Monte Carlo settings
☒	☐	For hierarchical and complex designs, identification of the appropriate level for tests and full reporting of outcomes
☒	☐	Estimates of effect sizes (e.g. Cohen's *d*, Pearson's *r*), indicating how they were calculated

Our web collection on statistics for biologists contains articles on many of the points above.

Software and code

Policy information about availability of computer code

Data collection	We used Phylophlan 3.0 (https://huttenhower.sph.harvard.edu/phylophlan/), Plasflow 1.1 (https://github.com/smaegol/PlasFlow) and the Comprehensive Antibiotic Resistance Database (https://card.mcmaster.ca/) in this study.
Data analysis	Analysis code (python and R) is available at https://github.com/Riselya/Plasmid-Project, which also includes an Rmarkdown analysis summary PDF outlining analysis steps from after the point the bacteria-plasmid association table was generated.

For manuscripts utilizing custom algorithms or software that are central to the research but not yet described in published literature, software must be made available to editors and reviewers. We strongly encourage code deposition in a community repository (e.g. GitHub). See the Nature Research guidelines for submitting code & software for further information.

Data

Policy information about availability of data

All manuscripts must include a data availability statement. This statement should provide the following information, where applicable:

- Accession codes, unique identifiers, or web links for publicly available datasets
- A list of figures that have associated raw data
- A description of any restrictions on data availability

Data used in this study was downloaded from the public data repository https://osf.io/ezb8j/ (uploaded as part of Stalder, T., Press, M. O., Sullivan, S., Liachko, I., & Top, E. M. (2019). Linking the resistome and plasmidome to the microbiome. The ISME journal, 13(10), 2437-2446.)

Field-specific reporting

Please select the one below that is the best fit for your research. If you are not sure, read the appropriate sections before making your selection.

☐ Life sciences ☐ Behavioural & social sciences ☒ Ecological, evolutionary & environmental sciences

For a reference copy of the document with all sections, see nature.com/documents/nr-reporting-summary-flat.pdf

Ecological, evolutionary & environmental sciences study design

All studies must disclose on these points even when the disclosure is negative.

Study description	We characterised a bacteria-plasmid network from one wastewater sample, generated by Stalder et al. 2019, ISME. Because Hi-C ligation technology was used during sequencing, host and plasmid DNA occupying the same cell can be identified. Stalder et al. (2019) made the bacterial metagenome -assembled genomes (MAGs) available, as well as the Hi-C connected DNA fragments. From this available data, we identified likely plasmid DNA fragments using the software Plasflow, then summarised which bacterial host they were connected with to using the Hi-C links data. The output was a host-plasmid association table, from which we characterised the network described in the publication.
Research sample	We used one wastewater sample generated by Stalder et al. 2019, which is available to download at https://osf.io/ezb8j/. The DNA contained in this sample was sequenced using Hi-C technology, which ligates DNA lying in close proximity (ie within the same cell). This method is fully explained in Stalder et al. 2019.
Sampling strategy	We characterize a bacteria-plasmid network from just one sample, therefore sampling strategy is not applicable.
Data collection	We downloaded the Hi-C sequence reads from one wastewater sample, made available by Stalder et al. 2019. The wastewater sample was collected in October 2017 at the Moscow WWTP in Idaho (USA). The facility services ~25,000 people and collects mainly domestic wastewater.
Timing and spatial scale	The wastewater sample was collected in October 2017 at the Moscow WWTP in Idaho (USA).
Data exclusions	We included only bacterial MAGs that had over 50% completeness (n = 191) in the network, since MAGs with low completeness would bias the study (since plasmid DNA is less likely to be detected). We also only analysed bacteria-plasmid connections that were recorded at least 50 times, ,to ensure these connections were robust and not due to sequencing error.
Reproducibility	We characterize a bacteria-plasmid network from one wastewater sample, and this network is available to be reproduced by anyone.
Randomization	We characterize a network from just one sample, therefore randomisation of samples is not applicable.
Blinding	We characterise a network from just one sample, therefore blinding of samples is not applicable.

Did the study involve field work? ☐ Yes ☒ No

Reporting for specific materials, systems and methods

We require information from authors about some types of materials, experimental systems and methods used in many studies. Here, indicate whether each material, system or method listed is relevant to your study. If you are not sure if a list item applies to your research, read the appropriate section before selecting a response.

Materials & experimental systems

n/a	Involved in the study
☒	☐ Antibodies
☒	☐ Eukaryotic cell lines
☒	☐ Palaeontology and archaeology
☒	☐ Animals and other organisms
☒	☐ Human research participants
☒	☐ Clinical data
☒	☐ Dual use research of concern

Methods

n/a	Involved in the study
☒	☐ ChIP-seq
☒	☐ Flow cytometry
☒	☐ MRI-based neuroimaging
